# *Photorhabdus* spp.: An Overview of the Beneficial Aspects of Mutualistic Bacteria of Insecticidal Nematodes

**DOI:** 10.3390/plants10081660

**Published:** 2021-08-12

**Authors:** Mahfouz M. M. Abd-Elgawad

**Affiliations:** National Research Centre, Plant Pathology Department, Agricultural and Biological Research Division, El-behooth St., Dokki, Giza 12622, Egypt; mahfouzian2000@yahoo.com

**Keywords:** biocontrol, *Heterorhabditis*, pest and pathogen management, *Photorhabdus*, marketing

## Abstract

The current approaches to sustainable agricultural development aspire to use safer means to control pests and pathogens. *Photorhabdus* bacteria that are insecticidal symbionts of entomopathogenic nematodes in the genus *Heterorhabditis* can provide such a service with a treasure trove of insecticidal compounds and an ability to cope with the insect immune system. This review highlights the need of *Photorhabdus*-derived insecticidal, fungicidal, pharmaceutical, parasiticidal, antimicrobial, and toxic materials to fit into current, or emerging, holistic strategies, mainly for managing plant pests and pathogens. The widespread use of these bacteria, however, has been slow, due to cost, natural presence within the uneven distribution of their nematode partners, and problems with trait stability during in vitro culture. Yet, progress has been made, showing an ability to overcome these obstacles via offering affordable mass production and mastered genome sequencing, while detecting more of their beneficial bacterial species/strains. Their high pathogenicity to a wide range of arthropods, efficiency against diseases, and versatility, suggest future promising industrial products. The many useful properties of these bacteria can facilitate their integration with other pest/disease management tactics for crop protection.

## 1. Introduction

In the agricultural sector, the current production practices are not sufficient to control insect pests and pathogens safely and with 100% efficacy. Growing dissatisfaction with the chemicals that are used for plant pest control has increased, due to their negative impacts on human health and non-target organisms, contamination, toxicity to the environment, and resistance development [1,2,3]. Thus, it has become imperative to decrease the use of these unsafe pesticides and replace them with benign ecologically sound products for crop pest control, in the context of sustainable agriculture.

Recently, the importance of using beneficial bacteria in integrated pest and pathogen management programs has been emphasized. These bacteria can reduce the chemical inputs used for plant protection, and stabilize ecological changes [4]. Conceivably, entomopathogenic bacterial species of the genus *Photorhabdus* (Enterobacteriales: Morganellaceae) may be a favorable alternative for expanding the biocontrol of many plant pests and pathogens, via their secretion of various arrays comprising effective bioactive metabolites [2,5,6,7,8,9]. This concept is based on a huge number of *Photorhabdus* genes that encode for producing relevant compounds, e.g., enzymes, toxins, antibiotics, and bacteriocins. They are found in the form of pathogenicity islands on the bacterial chromosomes representing different groups, e.g., the group of toxins is assorted to four prime groups. Additionally, discoveries of novel species of *Photorhabdus*, with presumed additional genes encoding for beneficial traits, are being discovered, and novel genes in the *Photorhabdus* species that are already described are ongoing, and can further be exploited [1,8,9,10,11]. The goal of this paper is to provide an overview on the pros and cons of *Photorhabdus* spp. as an all-in-one resource for biocontrol. The aim is not only to focus on the related background of the state-of-the-art knowledge, but also to highlight the modern biotechnology that can open further avenues of discovery that can be leveraged towards enhancing sustainable agriculture. So, this review addresses recent findings on their diversity, new taxa, and use in the existing or emerging programs to manage plant pests and pathogens.

## 2. Taxa, Diversity, and Lifestyle of *Photorhabdus* spp.

### 2.1. Their Taxa and Diversity

The number of *Photorhabdus* species has recently doubled, from four [12] to twenty [13]. An updated list of *Photorhabdus*, comprising the valid species/subspecies, is presented (Table 1). Yet, they are expected to increase as, in addition to their importance, much focus is directed to employ morphological, biochemical, physiological, and molecular approaches to characterize and identify them as mutualistic/symbiotic bacteria of *Heterorhabditis* spp. Some of *Photorhabdus* spp. are further divided into subspecies (Table 1). Using the entire genome-established phylogenesis of the *Photorhabdus* taxa (species, subspecies, strains, and isolates), a novel phylogenetic tree of the genus *Photorhabdus*, with new taxa, has been reconstructed [13]. For instance, considering the recent sequence comparative investigations of the related taxa, *P*. *aegyptia* was erected as a new species. Its type strain is a symbiont of *H. indica*, originally collected from Egypt.

As heterorhabditid nematodes are found in many, and wide, geographical areas, their symbionts, *Photorhabdus* spp., must have global distribution. An overall heterorhabditid nematode geographic distribution was reviewed [5]. While the most widespread species, *H. bacteriophora*, is found in zones having continental and Mediterranean climates, *H. indica* is familiar to the subtropics and tropics. *Heterorhabditis megidis* generally has a more northerly and more limited distribution than *H. bacteriophora*. The EPN species are found worldwide; the only continent where these nematodes have not been found is Antarctica. The longstanding recognition concerning the species-specific identification for the complex of *Photorhabdus*-*Heterorhabditis* as a dyad in their mutualism, is still generally valid. Thus, it can gain more knowledge regarding their distribution in space and diversity [14].

Basically, researchers have been trying to optimize EPN surveys and extraction methods [15,16] to discover novel species/strains that have adapted to local conditions, and to boost the effective control of pests and pathogens. Therefore, it can be generally assumed that the distribution of the EPN species is just an artefact of such sampling trials. However, increased attention is mainly paid to these bacteria when applied to kill insect pests and pathogens independently of their EPN partners.

Eventually, fueled by the gradual increase in the number of new *Photorhabdus* species/strains and novel technological approaches to identify them, the *Photorhabdus* taxa have become clearer and easier to study than before. Therefore, complete genome-constructed phylogenetic trees, along with accurate sequence comparative investigations, could correctly determine their relationship and diversity. This identification could also contribute to additional investigations of the relevant *Photorhabdus* spp.-bioactive compounds that can be applied in industrial and agricultural products [2,13,17].

**Table 1:** Updated list of species/subspecies in the genus *Photorhabdus* and their mutualistic heterorhabditid nematodes.

*Photorhabduss* Species	*Heterorhabditis* Species	References
*P. akhurstii*	*H. indica*	[18,19]
*P. asymbiotica*	undescribed species	[18,20]
*P. australis* subsp. *Thailandensis* subsp. *australis*	*H. gerrardi*, *H. indica*	[13,19,20]
*P. bodei*	*H. beicherriana*	[19]
*P. caribbeanensis*	*H. bacteriophora*	[19,21]
*P. cinerea*	*H. downesi*, *H. megidis,* *H. bacteriophora*	[19,22]
*P. hainanensis*	undescribed species	[19,21]
*P. heterorhabditis* subsp. *Aluminescens* subsp. *heterorhabditis*	*H. zealandica*	[13,23]
*P. kayaii*	*H. bacteriophora*	[19,24]
*P. khanii*	*H. bacteriophora*	[19,21]
subsp. *guanajuatensis*	*H. atacamensis*	[25]
*P. kleinii*	*H. georgiana*, *H. bacteriophora*	[19,26]
*P. laumondii* subsp. *clarkei* subsp. *laumondii*	*H. bacteriophora*	[18,19]
*P. luminescens*	*H. bacteriophora*, *H. indica*	[27,28]
subsp. *sonorensis*	*H. sonorensis*	[29]
subsp. *mexicana*	*H. mexicana*	[25]
*P. namnaonensis*	*H. baujardi*	[19,30]
*P. noenieputensis*	*H. indica*, *Heterorhabditis* sp.	[19,31]
*P. stackebrandtii*	*H. bacteriophora*, *H. georgiana*	[19,32]
*P. tasmanensis*	*H. zealandica*, *H. marelatus*	[19,21]
*P. temperata*	*H. megidis*, *H. downesi,* *H. zealandica*	[18,19]
*P. thracensis*	*H. bacteriophora*	[19,21,24]
*P. aegyptia*	*H. indica*	[13]

### 2.2. The Lifestyle of Photorhabdus spp.

All the species of *Photorhabdus* are exclusively found as symbionts of the *Heterorhabditis* spp.-infective juvenile (IJ) stage [33]. As these bacteria have not been detected in free-living form in nature, they had previously raised doubts of their competence to live and infect insect pests and pathogens in the absence of their EPN partner. An exception is *P. asymbiotica*, which is known to be a human pathogen, although it also infects insects [34]. This species causes ulcerated skin lesions, both at the initial infection foci and, later, at disseminated distal sites [35]. Another featured difference to the other *Photorhabdus* spp. is the typical coloration of grey with pink spots, acquired by the *P. asymbiotica*-infected insect host, but that color for the other species of *Photorhabdus* is usually reddish [36].

During mutualism, these bacteria offer a few favors to the heterorhabditid nematodes, in terms of killing insects that are invaded by the nematodes, and supplying nutrition and defense to these entomopathogenic nematodes within the infected cadavers. The nematodes pay back such favors by protecting the mutualistic bacteria outside their insect host from harsh conditions, while enabling them to set off and multiply within the attacked insect hosts. Notwithstanding the EPNs, as originally soil-inhabiting creatures, their free-living stage, as IJ, harbors the bacteria in its intestinal lumen [33]. Due to their tripartite association [37], *Photorhabdus* spp. are naturally found either in their infected insect hosts or at the intestinal lumen (gut mucosa) of their nematode partner. As the IJs penetrate their insect hosts via natural openings (spiracles, anus, or mouth) or the cuticle, they move forward to the haemocoel, where they let out their mutualistic bacteria to multiply and produce secondary metabolites that kill their infected arthropods and turn their bodies into a nutrient soup. During this bioconversion, the nematode feed on the soup, in order to develop and reproduce while the bacteria produce antibiotics that keep the infected insect away from further microbial attacks.

Referring to *Photorhabdus* is frequently accompanied by its counterpart *Xenorhabdus* bacteria, because there is a great similarity between them in the general framework of living. *Xenorhabdus* and *Photorhabdus* are symbionts of EPN-IJs, of the genera *Steinernema* and *Heterorhabditis*, respectively. However, each of the two bacterial genera has its own attributes that distinguish its character. *Xenorhabdus* spp. live within a specialized receptacle at the front portion of the intestine, but *Photorhabdus* spp. are set at the gut mucosa of their respective nematodes [38,39]. The IJs of *Steinernema* spp. develop into amphimictic females and males. So, both sexes must infect the insect host and mate to reproduce in the insect hemocele, but the *Heterorhabditis*–IJs mature to hermaphroditic females and then to the two sexes. Thus, a single hermaphroditic *Heterorhabditis*–IJ can infect and multiply within the insect host. Also, the colonization behavior of the two bacterial genera differs within their nematode partners [39]. A significant trait that is used to distinguish between the two EPN genera, in case of their presence within their insect host, is the pigment of the *Photorhabdus* bacterium to color the host’s body in a reddish shade. *Photorhabdus* is capable of fluorescing so often that the entire infected cadavers glow in unlighted places. *Photorhabdus* avoid the immune system of the insect host by adjusting the lipopolysaccharide to withstand the impact of the insect-derived antimicrobial produce of peptides, while *Xenorhabdus* disturbs the induction of such peptide expression [38]. Their 16S rRNA genes are more than 94% identical, though the genome of each genus may be disrupted by numerous deletions, inversions, insertions, and translocations [40]. *Photorhabdus* bacteria can go through major transcriptional reforming in the intestine of their EPN partner. They can induce general starvation mechanisms, turn into the pentose phosphate pathway to cope with oxidative stress and nutrition deficit, cellular acidification to slacken growth, and form biofilms to safely remain in the EPN intestine until transmitting to the insect hemolymph [32]. Such bacterial strategies can sustain them within the nematode gut and ensure felicitous transmission of the couple from one insect host to another. Interestingly, the *Xenorhabdus* and *Photorhabdus* species are able to grow in vitro as free-living organisms, without their partner EPNs, on artificial media under certain terms, i.e., adequate nutrient media with no competition.

Until the beginnings of the current century, it was believed that the relationship between EPNs and their mutualistic *Photorhabdus* bacteria was extremely specific, i.e., each EPN species carries only a corresponding bacterial species or subspecies. Contrary to the current findings [41,42,43], it was thought that the tight relatedness of the two taxonomic groups conduces co-speciation between each pair of *Photorhabdus* spp. and *Heterorhabditis* spp., i.e., the mutualism between *Heterorhabditis* and *Photorhabdus* had been previously thought to be strictly one-to-one, in terms of co-speciation [33,44]. However, an opposite opinion is exemplified in the cohabitation of mixed *Photorhabdus* species (*P*. *cinerea* and *P*. *temperata*) within a single nematode host; *Heterorhabditis downesi* [45]. The latter authors could further examine the aspects of competition between *P*. *cinerea* and *P*. *temperata*, associated with *H*. *downesi* at two levels. Apparently, *P*. *cinerea* can better protect the nematode against desiccation at the regional level, though *P*. *temperata* is superior to *P*. *cinerea* in protecting the scavengers that utilize vision in foraging. Moreover, *P*. *cinerea* surpasses *P*. *temperate* at the local level/within the infected insect host.

## 3. Pathogenicity of *Photorhabdus* spp.

### 3.1. Range and Magnitude of Pathogenicity

Basically, various *Heterorhabditis*-*Photorhabdus* partnerships, to infect and kill many insect pests, have been commercially applied as biocontrol agents, with continuous suggestions for developments, to prime them for effective alternative measures in plant protection [37,46,47,48]. In such cases of the natural EPN–bacterium complex, the bacterial host range is naturally confined to the ability of the IJs to find and penetrate the host, as a pre-requisite for the exponential growth of *Photorhabdus* spp. to attain high cell densities within the insect host. The bacterial cells can convert the insect tissues into a biomass that is necessary for the IJ development and reproduction. The pathogenicity relies on the bacterial growth. Therefore, the *Photorhabdus* growth rate is closely correlated with the time that is required for killing the insect. Admittedly, *Photorhabdus* spp. are highly virulent pathogens of a wide range of insect larvae [49].

**Figure 1:**
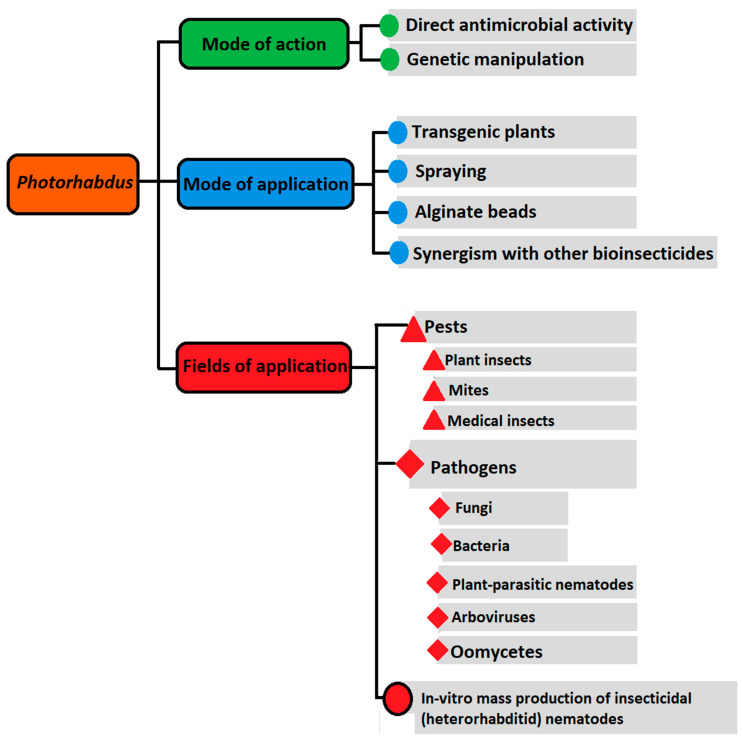
Simplified illustration for possible scopes of application, mode of application and mechanism of action of *Photorhabdus* bacteria to control various pests and pathogens.

Detecting the ability of *Photorhabdus* bacteria to survive in soil and in fresh water for 1 week, has probably fixed a time frame for their additional biocontrol applications, independent of their mutualistic EPNs [50]. Hence, various formulations (Figure 1), mainly based on just the bacteria and/or bacterial metabolites, have been recorded [2,4,12,51,52,53,54,55,56,57]. In this regard, increased pathogenicity islands of the *Photorhabdus* chromosome, with many genes encoding various insecticidal protein toxins, antibiotics, bacteriocins, and enzymes, were reviewed [5,58,59], but more have still been further identified, e.g., [55,56,60,61]. For instance, the insecticidal categories of protein toxins comprise toxin complexes (TCs), *Photorhabdus* insect-related (Pir) proteins, makes caterpillars floppy (Mcf) toxins, *Photorhabdus* virulence cassettes (Pvc), *Photorhabdus* insecticidal toxin (Pit), Photox, PaxAB, and Galtox [9]. While TCs are large multi-component toxins, others, such as Photox, are a smaller 46 kDa binary.

Clearly, the above-mentioned traits of the bacteria—such as the ability to circumvent the insect’s immune system, reforming and adapting to the surroundings, especially under stressed conditions, and worldwide spread with a treasure trove of useful compounds—are compelling for their relevant usage. They may be a rationale to reflect their capacity in the broad and efficient biocontrol of plant pests and pathogens. Their impressive array of primary and secondary metabolites may be so virulent that a single bacterial cell is enough to kill the targeted host of some arthropods, within a relatively short period of time [49]. *Photorhabdus* bacteria are, independently, quite eligible to control a broad range of arthropod pests in numerous categories, especially in the orders Lepidoptera (butterflies and moths), Diptera (flies, including insects that transmit human and plant diseases), and Coleoptera (weevils and beetles) [2,7,62]. For instance, *P*. *luminescens* and its metabolites were found to be lethal to the greater wax moth, *Galleria mellonella*, when applied in sand media; the bacterium rapidly penetrated the *G. mellonella* hemocoele as it got contact with the larval body. Its toxic metabolites caused more larval death than the *P. luminescens* cells [63]. As the bacterial cells do have a free-living existence and can enter the insect haemocoele in the absence of the EPN vector, those authors stressed that the bacterium, or its toxic secretions, can be used for insect control, as an important component of various integrated pest management (IPM) programs.

Yet, *P. luminescens* received much research work, due to the type of species of its genus, with a worldwide distribution and high insecticidal activities. Such activities against the diamondback moth, *Plutella xylostella*, pupae surpassed that of *X. nematophila* [64]. The two bacterial species induced 60% and 40% mortality of *P. xylostella* pupae, with LC_50_ values of 5 × 10^4^ and 5.5 × 10^5^ cells/mL, respectively. The 48-h LC_50_ for toxin complex a (Tca) of *P. luminescens* against neonates of the Colorado potato beetle, *Leptinotarsa decemlineata*, was 2.7 ppm, and the second instar larval growth that was exposed to Tca for 72 h was almost fully inhibited at >0.5 ppm [65]. The strain K-1 of *P. luminescens akhurstii,* encapsulated in sodium alginate beads (2.5 × 10^7^ cells/bead) and mixed with sterilized soil, killed 100% of the tobacco cutworm, *Spodoptera litura*, larvae in 48 h, though its partner (i.e., *H. indica*–IJs that possess the bacteria in their gut) scored only 40% mortality after 72 h. Koch’s postulates for this strain, without the symbiont nematode, were evident, as the bacteria could be re-isolated from the dead insect. The LC_50_ dose of the strain K-1 was 1010 cells per *S. litura* sixth-instar larvae in 48 h [53]. A 100% mortality of both the fall armyworms, *Spodoptera frugiperda* and *G. mellonella*, was also attained after 48 h of treatment with the strain *P. luminescens akhurstii* SL0708 at 1 × 10^3^–1 × 10^4^ CFU/larva [66]. On the other hand, *P. temperata* showed oral toxicity to the olive moth, *Prays oleae*, with an LC_50_ of 58.1 × 10^6^ cells/mL [67].

Mohan et al. [51] tested the potential of *P. luminescens* to control the cabbage butterfly (*Pieris brassicae*), which is a polyphagous pest of crops in the family Brassicae. The bacterial cultures were grown overnight, in nutrient broth at 28 °C, up to a concentration of 10^8^ colony-forming units (CFU)/mL. The bacterial culture was mixed with paraffin oil at 10 l/mL, Tween-20 at 0.5 l/mL, and sucrose at 0.5%, as a phagostimulant adjuvant using sterile water as the base before spraying. Initially, Mohan et al. [51] tested the survival and retention of bacterial pathogenicity in combination with various components used in the formulation. When the formulated *P. luminescens*, stored at 28 °C, was sprayed uniformly on the foliage of ornamental nasturtium, *Tropaeolum majus*, which was heavily infested with the 3–4 instar of *P. brassicae*, 100% mortality of the larvae was recorded within 24 h, compared to no mortality in the control plot. Re-isolation of the bacteria from the dead insects, and comparison with the original culture, proved Koch’s postulates. Such results proved the direct toxicity of *P. luminescens* to the insects, under natural conditions, when used as a foliar spray. Also, Jallouli et al. [68] recorded promising insecticidal activity of *P. temperate* K122 against a stored grain pest, the Mediterranean flour moth, *Ephestia kuehniella*. At a high concentration of 12 × 10^8^ cells/mL, 100% mortality of *E. kuehniella* larvae could be reached. Jallouli et al. [68] concluded that the insect mortality was due to toxaemia, as confirmed by the absence of variant small colonies, or *P. temperata* colonies, in *E. kuehniella* tissue. The histopathological effect of *P. temperata* toxins on the gut of infected *E. kuehniella* larvae indicated destruction of the gut epithelium, the appearance of large cavities, and cellular disintegration.

In addition to using *Photorhabdus*-concentrated metabolites or bacterial broth treatments, another avenue for pest or disease suppression is the development of bioactive compounds that are responsible for bacterial toxicity. Various active compounds in *Photorhabdus* spp. were reviewed [5]. For example, transcinnamic acid (TCA) was recently reported to be a major active compound in *P. luminescens*’ suppressive activity against *Fusicladium effusum*, and thus further research to develop TCA, as a potential control agent, was suggested [69]. More research on the identification and activity of such bioactive compounds is warranted. Furthermore, regardless of the type of treatment (bioactive chemicals, metabolites and/or bacterial treatments), field testing and economic feasibility analysis will be needed, as various biotic and abiotic factors outside the laboratory may reduce the potency and longevity of the tested materials.

It appears that the arsenal of these bacteria still contains much that has yet to be discovered, against a broad range of various pathogens. The detection and cloning of other beneficial compounds from *Photorhabdus* bacteria are still ongoing [11]. Eventually, the bacteria in this genus possess metabolites with the main characteristics of common pesticides, i.e., their effect increases with an increase in the dose, and a negative correlation exists between the number of eggs laid/insects that are female, percentage of hatching, adult survival of the pest, and the bacterial dose [5]. Their toxins are so fatal that as low as 40 ng is sufficient to kill the tobacco hornworm *Manduca sexta* larvae [70].

### 3.2. Mode of Action of the Bacteria and Their Secreted Compounds

These bacteria are commonly known to kill insects via septicemia/toxemia, within the context of the natural *Photorhabdus*–*Heterorhabditis* complex [54]. However, stand-alone pathogenicity tests of the bacteria and/or their secreted compounds usually begin with injecting them directly into the insect haemocoel, by artificial means [71]. Bacterial protein toxins usually have oral and/or injectable toxicity to insects, with various modes of action [72]. The increasing ambition to exploit *Photorhabdus*-derived compounds in industry is due not only to their abundance, but also to their qualities that boost their functions. Complete genome-sequencing investigations, which started in 2003 using *P*. *luminescens* as a model [73,74], have been revealing the capacity of various *Photorhabdus* spp. to produce numerous secondary metabolites, such as peptides, polyketides, toxins, and hybrids. For instance, the parasiticidal material that is derived from *P*. *luminescens* metabolites is recognized as a small molecule that is stable at both pH changes in the range 2–12 and heating. This molecule can induce the trypanocidal activity via a mode of action that does not rely on nitric oxide [56]. Likewise, *P*. *luminescens* materials, such as isopropylstilbene [75] and the presumed GameXPeptides [76], demonstrated anti-*Leishmania donovani*, -*Trypanosoma cruzi*, and -*Plasmodium* leverage, respectively [55].

Each of the above-mentioned categories of toxins has a possible function as a biocontrol material, via a specific mechanism against arthropod pests, pathogens, and/or vector insects. While the Tcs damage epithelial cells at the insect intestine, for instance, Mcf enhances hemocytes apoptosis in the insect hemocoel [77]. On the other hand, Pvc can induce *G. mellonella* and *Manduca sexta* mortality, but Pir proteins of *P. luminescens laumondi* (TT01 strain) are responsible for insect death [2]. As Pvc can show a self-contained nanosyringe delivery mechanism, it can beat host cell membrane barriers and function independently from its bacterium, to disrupt the cytoskeleton of the insect host [78]. Some *Photorhabdus* species can poison the intestinal epithelium of their insect hosts, via high-molecular-weight Mcf toxin. It can enable *Esherichia coli* both to persist within, and kill, the insect [79]. Thus, these toxins show bacterial strain-specific variations concerning toxicity to their insect hosts [9,80]. Different features regarding the detailed structure, mode of action, and putative function of the Tcs as ‘polymorphic’ toxins in the process of infection, have been discussed [81,82], but the modes of action of some recently detected metabolic compounds of *Photorhabdus* bacteria still need to be grasped, to enable their perfect use in the management of agricultural pathogens and insect pests [8,9]. *Photorhabdus* bacteria can also suppress important endoparasitic nematode species within plant roots, via their toxins and antibiotic compounds [5]. Furthermore, the Tc toxins could be cloned into *Arabidopsis* as a model plant, in order to offer protection from arthropod pests (Figure 1). Thus, these toxins are proposed as a potential substitution to the Bt toxin [49], for which insect resistance is developed [1].

The many examples of arthropod pest and pathogen mortalities that are caused by *Photorhabdus* spp. [3,5,48,53,83,84,85], do not negate the differences in the immune response between insect hosts. For instance, a cumulative percent mortality of 63% and 100% for *G. mellonella,* but 10% and 93% for *S. frugiperda,* was achieved after 72 h of injecting intra- or extra-cellular extracts of the strain *P. luminescens akhurstii* SL0708, respectively. These differences also indicate the impact of extracellular factors in pathogenicity [66]. Those authors detected proteases, esterases, ureases, hemolysins and siderophores as responsible for the high pathogenicity/extra-cellular activities. Moreover, the variation in immune response between host species may be due to both the evolutionary/environmental and biologic/genetic factors that are assigned to each host–pathogen system. The different system components, comprising induction, specificity, and memory of the immune system, can define the cognate resistance mechanism of the targeted insect species/population [86]. Abd El-Zaher et al. [87] reported that physical parameters (i.e., temperature, pH, and sodium chloride) variably affected the metabolite-induced mortality percentage for the *G. mellonella* larvae. Shapiro-Ilan et al. [88] reported that implementations of the bacteria and fermentation broth, to suppress pecan and peach pathogens, could be useful in reducing costs and/or avoiding regulatory issues compared to applying concentrated metabolites of these bacteria. However, they reported that the applications of bacterial broth could only inhibit the lesion growth that is induced by *Phytophthora cactorum* of pecan leaves, but its efficacy to the two other pathogens examined (*F. effusum* and *Armillaria tabescens*) was not apparent. Therefore, they perceived that more broth rates would stimulate a response. Alternatively, the toxicity of active compounds within the bacterial cell suspension, broth, or cell-free filtrates could be enhanced through medium optimization (e.g., [87,89]).

## 4. Pros and Cons of *Photorhabdus* spp.

### 4.1. The Positive Aspects

The above-mentioned common issues that are related to many chemical pesticides, as well as the costly and/or mixed performance of numerous existing biopesticides on one hand and the high levels of *Photorhabdus* virulence towards a wide variety of insects via just a few bacterial cells on the other, have been attracting commercial attention to use these bacteria and their bioactive compounds as new biopesticides [9,12,52,54]. Remarkably, the bacteria’s recent inexpensive in vitro mass production [90] should increase the interest of many researchers in related fields (microbiology, nematology, molecular biology, pharmacology, etc.), for their beneficial applications. Collectively, *Photorhabdus* bacteria can be adopted for large-scale application, due to some of their characteristics discussed in the following section.

#### 4.1.1. Cost-Effective Photorhabdus Mass Culture with Boosting Insecticidal Activity

In the recent past, many difficulties in the trait deterioration of *Photorhabdus* spp. were apparent during their in vitro mass culture. Furthermore, researchers are challenged by a cost and benefit tradeoff. Reduced costs with high bacterial yield, in terms of the scale-up of the mass production, are opposed by the requirement, to preserve beneficial *Photorhabdus* traits during the process of culturing [91]. Such bacterial attributes are significant for biocontrol programs, whether via *Photorhabdus* alone or the EPN–*Photorhabdus* complex. Fortunately, these bacterial traits could be characterized and maintained during their growth and metabolic phases of their inexpensive culturing [90]. Consequently, the full *Photorhabdus* capacity could be employed for the insect pathogenicity. Some significant advances in in vitro culture have been made. For example, Orozco-Hidalgo et al. [92] found that bacterial inoculation of the culture for 36 h could offer the highest *H*. *indica*–IJ yield. The authors stressed the merit of glucose as a substrate to increase/sustain the bacteria, so that higher nematode recovery could be achieved. Recently, an important discovery offers potentially the best conditions for enhancing the mass culture and the insecticidal efficacy of *P. temperata,* using the wastewater of the food industry as a basis for the medium [90]. The authors used both a special layout (Box–Behnken design) and response surface methodology (RSM) to optimize the bacterial mass production. This layout was based on three factors, carbon/nitrogen (C/N) ratio, sodium chloride concentration, and bacterial inoculum size. Both insecticidal efficacy and bacterial mass culture were tied to the media parameters. Furthermore, when wastewater from the food industry was optimized for utilization, as an inexpensive raw material for *P*. *temperate* culturing, its production costs were only USD 35 per kilogram medium [90], compared to the USD 679 per kilogram medium that used yeast extract and glucose as the main components [93]. About a 95% reduction in the total production cost was achieved, while the produced bacteria had high insecticidal activity [90]. Thus, this technique for optimizing the important combined factors via RSM, to yield a superior bioinsecticide with high bacterial mass production in a short time (48 h incubation), should be expanded to other species of *Photorhabdus*. This inexpensive technique should be considered for large-scale practice, as it also gives a share in getting low-cost formulations that are able to compete with conventional chemical compounds.

#### 4.1.2. The Mounting Role of *Photorhabdus* Bacteria against Pests and Pathogens

The bacteria and their active metabolites show other merits that bode well for inclusion in promising approaches of modern farming. Consequently, broad biocontrol application is feasible against insects, fungi, oomycetes, mites, and bacteria infecting plants and, to a lesser degree, animals. Some examples of the expanding utility of *Photorhabdus* spp. include the following: (1) many *Photorhabdus* bacterial genes encode metabolites and toxins mostly with low molecular weight. These toxins proved to have insecticidal [94,95], antifungal, antibiotic [96], and antiparasitic [55,56] activities. They are effective, for instance, against the mushroom mite *Luciaphorus* sp. [97], *Venturia effusa* (a fungus causing pecan scab) [69,98], oomycetes that can severely limit the commercial productivity of pecans, peaches, and other fruit and nut trees [7,84], antibiotic-resistant bacteria [8], and even vector insects of human diseases, such as the mosquito species *Aedes aegypti* and *Ae. albopictus* [2]. Further, da Silva et al. [2] have reviewed the dengue virus as the most serious arbovirus, in reference to human morbidity and mortality. The viral serotypes can be transmitted mainly by females of *Ae. aegypti* mosquitoes, where the toxic effect of Cry4Ba that is derived from Bti against *Ae. Aegypti* could be enhanced by the *Xenorhabdus* and *Photorhabdus* bacteria; (2) ongoing field and laboratory assays can offer these bacteria and their nematode partners new positions for upgrading the control of additional pests via IPM programs [37,48,99,100]. Also, six *P. luminescens* isolates had antibacterial activities; each against one, two, or three of the tested bacterial species [101]. The metabolites or cell filtrates of some *Photorhabdus* isolates may have superior toxicity, relative to others tested as well [84]; (3) *Photorhabdus* species possess various toxins and compounds with various modes of action and sophisticated secretion systems that offer specificity of the cell surface receptor to dictate a specific interaction between the bacterial toxin and the insect midgut [82]; (4) *Photorhabdus luminescens* is so effective against the diamondback moth, *Plutella xylostella,* pupae that its pathogenic capability is superior to *X. nematophila,* though both bacterial species have a convergent lifestyle [64]; (5) *Photorhabdus* species could operate more effectively against pests via additive or synergistic incorporation with other advantageous inputs, such as another biocontrol agent [102,103]; (6) the toxic secretion of *P*. *luminescens* can be efficiently used to control various challenging pest populations, such as *Galleria mellonella* and subterranean termite *Macrotermis* spp. [85]. Shahina et al. [85] speculated that commercial use of *P*. *luminescens* cell suspensions as pesticides may overrule the problems of cost and reliability that are linked to the large-scale application of EPNs; (7) some *Photorhabdus* spp. showed miticidal activities against the economically important spider mite *Tetranychus urticae*, where the mortality rate increased as the bacterial dose or time elapsed increased [6]; (8) the supernatants of the *P. luminescens* culture blocked the nourishment of crickets, ants, and wasps [83,104]; (9) *Photorhabdus* toxin genes can generate transgenic plants for insect resistance [105,106]. There is potential to commercially produce an orally active agent from *Photorhabdus* bacteria for insect-resistant transgenic plant species. The prolonged monoculture of Bt transgenic plant cultivars has led to developing insects that are resistant against these cultivars, and the emergence of unforeseen pest problems [1]. Hence, *Photorhabdus* bacteria are being firmly suggested as an adequate alternative for Bt [2,9]. However, progress toward transgenic *Photorhabdus*-based plants is slow, as the current Bt transgenic strategies remain globally prevalent. Technical problems are usually linked to expressing a large, multi-subunit protein toxin into the needed transgenic plants, but small toxins of *Photorhabdus* and *Xenorhabdus* bacteria are among the best alternative sources of such insecticidal protein toxins [14,74]; and (10) these bacteria can favorably interact with plant roots as well [60]. The roots may attract EPNs that carry these bacteria to help control plant pests and pathogens [107,108]. Bacterial suspensions of *P*. *luminescens*, *Xenorhabdus* sp., and *X. szentirmaii* could significantly reduce the growth parameters of the root-knot nematode *Meloidogyne hapla*. They decreased its reproduction factor (RF) (55–62%), egg masses (48–68%), and number of galls (51–67%). Likewise, the cell-free supernatant of these bacteria reduced the number of egg masses (72–83%), galls (51–74%), and RF (62–72%) of the false root-knot nematode, *Nacobbus aberrans* [4,109]. Interestingly, on many economically important crops, the costs of controlling plant parasitic nematodes with these inexpensively produced bacteria [90] would generally be more economical than using common chemical nematicides, such as Cadusafos and Oxamyl. This is evidenced by the reported costs of using such chemicals on tomatoes [110], pepper [111], potatoes [112], and eggplant [113]. In addition, the application of *Photorhabdus* bacteria may also score a two-fold goal, i.e., control of the plant pathogens and insect pests simultaneously. Yet, achieving such a two-fold goal will necessitate optimal application tactics to maximize the field effectiveness of the *Photorhabdus* bacteria, e.g., a delivery system that is most conducive for the favorable and effective control of crop pests and pathogens via biocontrol agents. For example, various media/additions were found to potentiate *Photorhabdus* cell suspensions and cell-free filtrates against insect pests [87,89]. Ultimately, these attributes should be fully exploited by applying them in conventional and organic farming systems.

#### 4.1.3. The Bacterial Metabolites as New Drugs for Diseases

Increased concern about controlling certain diseases has created much interest for safer and more effective drugs than the currently used ones. For instance, the only effective approach to control insect-borne diseases, such as Zika, chikungunya, Chagas, Leishmaniasis, and dengue, is to block the detrimental transmission of the disease-causal organisms. However, the control of these insect vectors has demonstrated issues, due to the expensive measures of their chemical and biological control. They frequently possess low specificity for the targeted vectors/organisms, and may also be toxic to non-target and beneficial organisms [2,56]. Additionally, evolved resistance was reported for common insecticides in global resistance-breaking populations of *Ae. albopictus* and *Ae. aegypti* [2]. Further, da Silva et al. [2], therefore, speculated that a broad variety of biologicals and chemicals should contribute to their control measure. They found entomopathogenic bacteria, e.g., *Photorhabdus* and *Xenorhabdus*, to rank high in this respect, as numerous studies have demonstrated their relevant efficiency. Within this frame, a peptide that is smaller than 3 kDa, secreted by *P*. *luminescens,* was aptly named as ‘*Photorhabdus*-derived leishmanicidal toxin’. This compound evidenced potent leishmanicidal action to suppress dimorphological forms (i.e., amastigote and promastigote forms) of *Leishmania amazonensis*. Leishmania-toxic peptide(s) could be new drugs concerning the remedy for leishmaniasis [55]. In another study, *Trypanosoma cruzi,* which gives rise to American trypanosomiasis (or Chagas disease of human beings) could be controlled via metabolites that are secreted by *P*. *luminescens* [56]. Recently, da Silva et al. [2] demonstrated the low specificity of the chemicals that are used against *Ae*. *aegypti* and *Ae*. *albopictus* that transmit diseases such as dengue, malaria, Zika, and chikungunya. On the contrary, they appreciated the value of toxin complexes and metabolites produced by *Photorhabdus* and *Xenorhabdus,* to effectively control such insect-borne diseases.

Some may mistakenly suspect that controlling these medical insects has no absolute, or even marginal, relationship with plant health. However, their related chemical insecticides, as the pyrethroids and organophosphate temephos, have become less effective, even against plant pests, due to their widespread use that includes these medical insects as well [2]. Thus, the non-use of such pesticides as organophosphates against medicinal insects will limit their over-application and avoid the pollution of the environment, plants, wildlife, and groundwater. Additionally, their non-excessive use may generally slow down the development of insect resistance.

#### 4.1.4. *Photorhabdus*-Derived Natural Compounds as a Source for Industrial Products

The above-mentioned paradigms of *Photorhabdus*-derived insecticidal, fungicidal, pharmaceutical, parasiticidal, antimicrobial, and toxic materials may still be expanded to represent various types of expected industrial products. The richness of the relevant interesting compounds is reflected by an increasing number of materials that have been, and are still being, identified from these bacteria [5,8,11,55,56,61,69,94,95,96,98]. The targeted testing for the bioactivity of molecules reflecting the bacterial secondary metabolite genes and gene clusters are reported to be scarce, or mostly focused on medical applications [114]. Specifically, the molecule indole caused high levels of paralysis of plant-parasitic nematodes, such as *Meloidogyne incognita* and *Bursaphelenchus* spp., at a concentration of 100 to 300 mg/mL. Stock et al. [114] reviewed these bacterial metabolites in terms of their nematicidal, antimycotic, antibacterial, and insecticidal activities Thus, such metabolites may set up an inexhaustible mine of useful compounds, with multiple activities for new industrial products, mainly for crop protection.

### 4.2. Avoiding Negative Aspects

As *Photorhabdus* bacteria are originally mutualistic of insecticidal nematodes and highly efficient insect pathogens, via myriad toxins and small molecule effectors, cautious strategies should be set up to integrate these bacteria and/or their bioactive compounds into effective and safe management programs of crop insect pests. For instance, while some insect pests have developed resistance against Bt toxin, employing *Photorhabdus* toxins with alternate modes of action may resolve the issues of developed insect resistance. Practical use of *P. luminescens*, in conjunction with *B. thuringiensis kurstaki,* inhibited the growth of the Egyptian cotton leafworm *Spodoptera littoralis.* This synergism between the two bioinsecticides could use *B*. *thuringiensis* as a delivery means for *Photorhabdus* bacteria to infect the *S*. *littoralis* hemocoel and to reduce the risk of developing insect resistance [102]. Since Bt toxin occupies 90% of the bioinsecticide market [5], materializing this example can significantly raise *Photorhabdus* marketing. Other economically important insect pests that develop Bt resistance comprise *Ostrinia nubilalis* (European corn borer), *Heliothis virescens* (tobacco budworm), *Pectonophora gossypiella* (pink bollworm moth), *Culex quinquefasciatus* (mosquito), *Ae. aegypti* (yellow fever mosquito), *Trichloro plusiani* (tiger moth), *Leptinotarsa decemlineata* (Colorado potato beetle), *Spodoptera exigua* (beet armyworm), and *Chryosomela scripta* (cottonwood leaf beetle) [115,116]. Moreover, the toxin complex protein, TcaA toxin, showed toxicity against a wide range of agricultural pests, though the phylogenetic tree that was erected for TcaA indicated that this toxin did not have any ancestral relationship with BT toxins [116].

If so, the anticipated transgenic plants will be regulated by relevant legislation to be certain that such plants with genes of *Photorhabdus* bacteria can be produced without health or environmental risks [36]. No resistance to these bacteria has been reported in insect populations so far, but this line of thinking should be followed with any relevant toxins, as a means to avert or at least delay the development of insect/pathogen resistance. Therefore, many more investigations should be conducted before the exclusive use of these bacteria or their metabolites as a commercial tool to control crop insect pests and pathogens. Studies that are needed to enable its wise utilization should address both fundamentals, such as their molecular structure and mode of action, and applied aspects, such as their environmental stability, toxicity of different bacterial species/isolates against various insect pests and pathogens, and safety against non-target organisms. For example, Kumar et al. [117] found that two *P. luminescens* isolates failed to infect both the diamondback moth and the oriental leafworm moth (*Spodoptera litura*). On the contrary, Abdel-Razek [64] and Rajagopal et al. [53] found other isolates of the same bacterial species to be effective against the diamondback moth and the oriental leafworm moth, respectively. Likewise, technical issues, regarding further stand-alone formulations of the bacteria, their shelf life, and application, should be searched, particularly for their adequate integration into IPM programs. Examples of their usage have been reported [51,52,68], but their expansion and documentation should be attempted in earnest. Notably, a culture broth of *P. temperate temperata* mixed with *B. thuringiensis tenebrionis,* named “Col-Kill”, proved efficacy in controlling the coleopteran *Phaedon brassicae* (Coleoptera: Chrysomelidae) [118], but it needs to be widely tested as a new, effective formulation. It could open another beneficial tactic of synergism and avoid the prolonged monoculture of Bt transgenic plants that have led to developing insect resistance.

Apart from the aforementioned concerns of *P. asymbiotica,* and occasional episodes of allergy processes that are experienced by people who have had close contact with *Photorhabdus* species and their symbiotic nematodes, these bacteria, similarly to EPNs, have a very low risk to human health [36]. Mohan and Sabir [119] and Fand et al. [120] reported other constraints, where *P. luminescens* harm *Trichogramma* parasitized insect eggs and do kill coccinellids (key mealybug predators), respectively. Therefore, the integration of the bacteria with other biocontrol agents should be utilized cautiously. Advances in biotechnological approaches, and consolidation of the *Photorhabdus* data with their regional and temporal efficacy and dynamics, may offer the holistic and sound knowledge that is needed to establish and evaluate the real benefits and risks of EPNs and/or their mutualistic bacteria in nature, in the long term. Given these basics, Abd-Elgawad [5] emphasized that management projects should be decided on a case-by-case basis for attaining the best *Photorhabdus* species or strain–pest (or pathogen) matching, without side effects to the fauna and flora of definite sites. For instance, the bacteria could be harmful to definite pollinators. *Photorhabdus* species may be able to digest the bee tissues effectively and offer a supply of nutrients to the mutualistic nematodes [121].

## 5. Conclusions

The potential of using *Photorhabdus* spp. as biocontrol agents in sustainable agriculture, against a broad range of insect pests and pathogens, is being boosted via various approaches. The discovery of novel species/strains worldwide will continue to broaden the pool of their bioactive compounds that are effective against economically important pests and pathogens. Developing inexpensive methods for their commercial production may expedite their use in the existing or emerging programs to manage plant pests and pathogens. Clearly, the toxins of these bacteria cause massive damage to the gut epithelium of insects, resulting in rupture of the gut integrity and crossing of the gut barrier. They could be used in foliar application or in the form of alginate beads; both as standalone insecticides for the most common management strategies. Furthermore, their long-acting strategies have been proved via incorporating their toxin genes into transgenic plants to control insect pests. *Photorhabdus*-derived insecticidal, fungicidal, parasiticidal, antimicrobial, and toxic materials should be leveraged to fit into holistic crop protection strategies. These may include their combination with other synergistic or additive compounds, to increase their efficacy while preventing, or reducing, the likelihood of developing pesticide-resistant pest/pathogen strains.

## Data Availability

Not applicable.
